# Molecular Evolution of the Nuclear Factor (Erythroid-Derived 2)-Like 2 Gene *Nrf2* in Old World Fruit Bats (Chiroptera: Pteropodidae)

**DOI:** 10.1371/journal.pone.0146274

**Published:** 2016-01-06

**Authors:** Qiuyuan Yin, Lei Zhu, Di Liu, David M. Irwin, Shuyi Zhang, Yi-Hsuan Pan

**Affiliations:** 1 Laboratory of Molecular Ecology and Evolution, Shanghai Engineering Research Center of Molecular Therapeutics and New Drug Development, East China Normal University, Shanghai, China; 2 State Key Laboratory of Genetic Resources and Evolution, Kunming Institute of Zoology, Chinese Academy of Sciences, Kunming, China; 3 Kunming College of Life Science, University of Chinese Academy of Sciences, Kunming, China; 4 Laboratory of Molecular Ecology and Evolution, Institute of Estuarine and Coastal Research, East China Normal University, Shanghai, China; 5 Department of Laboratory Medicine and Pathobiology, University of Toronto, Toronto, Canada; 6 College of Animal Science and Veterinary Medicine, Shenyang Agricultural University, Shenyang, China; 7 Laboratory of Molecular Ecology and Evolution, School of Life Sciences, East China Normal University, Shanghai, China; Vanderbilt University, UNITED STATES

## Abstract

Mammals developed antioxidant systems to defend against oxidative damage in their daily life. Enzymatic antioxidants and low molecular weight antioxidants (LMWAs) constitute major parts of the antioxidant systems. Nuclear factor (erythroid-derived 2)-like 2 (Nrf2, encoded by the *Nrf2* gene) is a central transcriptional regulator, regulating transcription, of many antioxidant enzymes. Frugivorous bats eat large amounts of fruits that contain high levels of LMWAs such as vitamin C, thus, a reliance on LMWAs might greatly reduce the need for antioxidant enzymes in comparison to insectivorous bats. Therefore, it is possible that frugivorous bats have a reduced need for Nrf2 function due to their substantial intake of diet-antioxidants. To test whether the *Nrf2* gene has undergone relaxed evolution in fruit-eating bats, we obtained *Nrf2* sequences from 16 species of bats, including four Old World fruit bats (Pteropodidae) and one New World fruit bat (Phyllostomidae). Our molecular evolutionary analyses revealed changes in the selection pressure acting on *Nrf2* gene and identified seven specific amino acid substitutions that occurred on the ancestral lineage leading to Old World fruit bats. Biochemical experiments were conducted to examine Nrf2 in Old World fruit bats and showed that the amount of catalase, which is regulated by Nrf2, was significantly lower in the brain, heart and liver of Old World fruit bats despite higher levels of Nrf2 protein in Old World fruit bats. Computational predictions suggest that three of these seven amino acid replacements might be deleterious to Nrf2 function. Therefore, the results suggest that *Nrf2* gene might have experienced relaxed constraint in Old World fruit bats, however, we cannot rule out the possibility of positive selection. Our study provides the first data on the molecular adaptation of *Nrf2* gene in frugivorous bats in compensation to the increased levels of LWMAs from their fruit-diet.

## Introduction

Free radicals, including reactive oxygen (ROS) and nitrogen species (RNS), are continuously produced by aerobic metabolism and from external exposure. Under normal physiological conditions, the major endogenous source of ROS is proton leakage from the electron transport chain (ETCs) in the mitochondria [1, 2]. Escaped protons react with oxygen, with approximately 1–2% of the oxygen consumed by cell being diverted to generate hydrogen peroxide [3] and superoxide (O_2_^**·-**^) [1], which are the primary ROS found in living cells. These reactive species can attack DNA, protein and other macromolecules leading to oxidative damage [4]. The uncontrolled generation of reactive species results in oxidative stress and is harmful to organisms as it causes pathological changes.

Organisms have developed antioxidant defense systems to protect against oxidative damage and maintain cellular homeostasis. Antioxidant defense systems consist of functionally connected groups of enzymes and low molecular weight antioxidants (LMWAs) [4]. Antioxidant enzymes, for example catalase and superoxide dismutase, are endogenously produced and are highly efficient at scavenging ROS. Transcription of genes for these enzymes is regulated by nuclear factor (erythroid-derived 2)-like 2 (Nrf2), a member of the cap ‘n’ collar (CNC) subfamily of basic region leucine zipper (bZip) transcription factors [5]. Nrf2 regulates the transcription of targeted genes via *cis*-acting enhancer elements known as the antioxidant response elements (AREs), located in the promoter regions, with the core sequence of the ARE being 5’-RTGAYNNNGCR-3’ [6, 7]. Nrf2 is ubiquitously expressed in tissues and is the main regulator of the redox response. Nrf2 knockout mice display substantially increased susceptibility to oxidative stress [8], and exhibit large decreases in the expression of catalase [9] and NQO1 [10]. On the other hand, some reactive species scavengers are not enzymes, but instead are LMWAs. LMWAs are a group of compounds that originate from endogenous sources or exogenously from diet, which are capable of eliminating reactive species in direct or indirect ways [4, 11]. Examples of diet-derived antioxidants are vitamins A, C and E and the carotenoids, which play major roles in mitigating the negative effects of reactive species [11].

Bats (order: Chiroptera) are one of the most widespread and diversified mammalian groups with over 1100 species in the world [12]. Like other mammals, bats also possess antioxidant defense systems to protect against damage caused by ROS. Numerous studies in bats have linked oxidative stress with longevity, with lower levels of radicals associated with longer life [13–15]. Antioxidants might have a role in this process, as bats are exceptional long-lived, compared with nonflying mammals of similar size and basal metabolic rate [15, 16]. Bats exhibit a great diversity in food habits including frugivory, nectarivory, insectivory, piscivory, carnivory and sanguivory [17, 18]. This diversity in food habits is considered to have evolved independently several times from insectivorous bat ancestors [19]. Old World fruit bats (OWFBs; Suborder: Yinpterochiroptera; Family: Pteropodidae) and New World fruit bats (NWFBs; Suborder: Yangochiroptera; Family: Phyllostomidae) evolved fruit and nectar-eating habits independently. However, some New World fruit bats switch their food preference to insects when fruits are less abundant [20]. Megabats, especially Old World fruit bats, feed only upon plants, fruits, and leaves. Marshall reported that nearly 188 kinds of plant genera are the food source of megabats, with 144 of them being fruits [21]. Bats belonging to Pteropodidae can ingest up to 2.5 times their body mass in fruit per night [22]. Fruits contain large amounts of vitamin C, α-tocopherol and β-carotene, molecules that are dietary antioxidants and play important roles in antioxidant systems [23]. It was also reported that the daily intake of fruits by *Artibeus jamaicensis* is more than 50% of its body weight and contained nearly 45mg ascorbate [24]. The GSH and α-tocopherol contents of frugivorous bats have been reported to higher than those of insectivorous bats [16]. Schneeberger et al. argued that LWMAs-rich diets might enable frugivorous bats to more efficiently remove pro-oxidants [25]. Danilo et al. studied antioxidative enzymes in five bats species, finding that the activities of catalase and SODs in frugivorous bats were lower than in insectivorous bats [16]. These results suggest that frugivorous bats have a lower reliance on antioxidative enzymes, as their decreased levels of enzymatic antioxidant systems are balanced through an increased dietary input of LMWAs. The finding of a lower levels of antioxidative enzymes in frugivorous bats [21], lead us to speculate that *Nrf2*, which is the central regulator of antioxidant enzyme gene transcription, may have undergone relaxed evolution in these bats.

To test our hypothesis, we sequenced the coding region of the *Nrf2* gene from 11 species of bats, which were then combined with the sequences from 5 other bat species databases. The molecular evolution of these sequences was then studied in bats and other mammals. To validate the results from our molecular evolutionary analyses, we investigated the expression levels of Nrf2 protein and a downstream antioxidant enzyme in representative species of frugivorous and insectivorous bats. While expanding our knowledge on antioxidant adaptations in frugivorous and insectivorous bats, our study might also provide additional insight into understanding the longevity of bats.

## Materials and Methods

### Ethics Statement

The Animal Ethics Committee of East China Normal University approved all procedures, including the collection of bats species and tissues (ID No: AR2012/03001). The capture of bats were permitted by the local Protection and Research Center and the locations of all samples are listed in S1 Table. None of the bat species used in this study are considered endangered. Animals used in this study were sacrificed by rapid decapitation after capture and all efforts were made to minimize potential pain and suffering.

### Animal and Tissue Collections

Details and capture locations of the bat species used in molecular cloning are listed in S1 Table. Neotropical bats used for molecular cloning were captured in Mexico during April, 2010, for a previous study [26]. Four representative bat species was adopted in the biochemical experiments, two insectivorous bats: *Myotis ricketti* (Suborder: Yangochiroptera), *Rhinolophus ferrumequinum* (Suborder: Yinpterochiroptera), and two frugivorous bats: *Rousettus leschenaultia* and *Cynopterus sphinx* (Old World fruit bats). Three male individuals from these of the four bats species were captured from different locations in China: *Myotis ricketti* from Fangshan Cave in Beijing (39°48'N, 115°42'E); *Rhinolophus ferrumequinum* from Fish Cave in Anhui Province (30°20'N, 117°50'E); *Rousettus leschenaultia* from Jinlun Cave of Mashan County in Guangxi Province (23°55'N, 108°26'E) and *Cynopterus sphinx* from Haikou Park in Hainan Province (20°02'N, 110°20'E), during August to September, 2013. Bats were immediately transported to the laboratory and housed in a large cage (150 cm × 180 cm × 200 cm) until they adapted to the new environment. For each of these species, three individuals were sacrificed by cervical dislocation under conditions that were as similar as possible. Tissues were sampled and immediately stored at -80°C until use.

### Isolation, Amplification and Sequencing of *Nrf2* Coding Sequences

Total RNA from 11 bats was isolated from brain tissue using TRizol reagent (Invitrogen) following the standard protocol. RNA was reverse-transcribed into cDNA using a SuperScript^TM^ Ⅲ Reverse Transcriptase Kit (Invitrogen). PCR reactions were conducted with primers designed within the coding region of published *Nrf2* cDNA sequences (S2 Table). Resulting PCR products were separated using a 1.5% agarose gel and purified with a Gel Extraction Kit (Qiagen), and then ligated into pGEM-T Easy vectors (Promega), with positive clones sequenced on an ABI sequencer (Applied Biosystems).

### Taxonomic Coverage

We sequenced most of the coding region of *Nrf2* gene from 11 bats species representing eight of the 17 chiropteran families. In suborder Yinpterochiropetra, we included two Old World fruit bats from family Pteropodidae (*Cynopterus sphinx*, *Rousettus leschenaultia*); we also included four insectivorous bats from the Yinpterochiropetra suborder: *Rhinolophus pusillus* (Rhinolophidae), *Hipposideros pratti* (Hipposideridae) and *Megaderma lyra* and *Megaderma spasma* (Megadermatidae). From the suborder Yangochiroptera, we included one New World fruit bat *Artibeus jamaicensis* (Phyllostomidae) and four insectivorous bats from three families: *Pteronotus parnelli* (Mormoopidea), *Myotis ricketti*, *Pipistrellus abramus* (Vespertilionidae) and *Taphozous melanopogon* (Emballonuridae). *Nrf2* genes from five additional species were retrieved from GeneBank, and included *Pteropus vampyrus* and *Pteropus alecto* (Pteropodidae), *Myotis brandtii*, *Myotis lucifugus* and *Myotis davidii* (Vespertilionidae). All new sequences were deposited into GeneBank with accession numbers KT345715-KT345725.

*Nrf2* sequences from five other mammal species were obtained from the Ensembl Database: *Homo sapiens* (ENSG00000116044), *Pan troglodytes* (ENSPTRG00000012677), *Canis familiaris* (ENSCAFG00000013506), *Bos taurus* (ENSBTAG00000019255), and *Ictidomys tridecemlineatus* (ENSSTOG00000000313). Details on all bats species and their corresponding *Nrf2* gene accession numbers are listed in S1 Table.

### Phylogenetic Reconstruction

*Nrf2* coding sequences from 16 bats and 5 other mammals were aligned using CLUSTAL W implemented in MEGA 6 with default setting [27]. First, we examined whether there was any evidence for recombination within our dataset, as recombination would have adverse effects on the power and accuracy of phylogenetic reconstruction and molecular evolutionary analyses [28]. We used GARD [29](from the Hyphy/Datamonkey web server [30]) with β–Γ rate variation and three and four rate classes to detect evidence of recombination breakpoints and test their statistical significance. The program jModelTest 0.1 [31] was used to evaluate the best nucleotide substitution model. A Bayesian phylogenetic tree was reconstructed based on the aligned nucleotide sequences using MrBayes 3.1.2 [32] with the GTR+G nucleotide substitution model, as selected by jModelTest 0.1. For the Bayesian analysis, ten million generations was set for the Markov chain, with the first two million discarded as burn in. The standard deviations of the split frequencies were stable below 0.01 after two million generations of MCMC. The MCMC was run in default setting of MrBayes 3.1.2. Datasets supporting the results are available in the Dryad digital repository, http://dx.doi.org/10.5061/dryad.kh186 (DOI:10.5061/dryad.kh186).

### Reconstruction of Ancestral Characters and Molecular Evolutionary Analyses

*Nrf2* coding sequence from 21 species used in this study were aligned by CLUSTAL W implemented in MEGA 6 [27] and checked for accuracy by eye. The alignment was then translated into amino acid sequences. In the process of sequence alignment, we identified seven amino acid changes that occurred in the Old World fruit bats. To trace the ancestral states for these lineage-specific amino acid changes, we assessed these substitutions using the maximum parsimony method with Mesquite 2.74 [33], the parameters set as defaults. For ancestral state reconstruction and selection tests, we used the phylogenetic tree topology based on the accepted species relationships [34–36].

**Fig 1:**
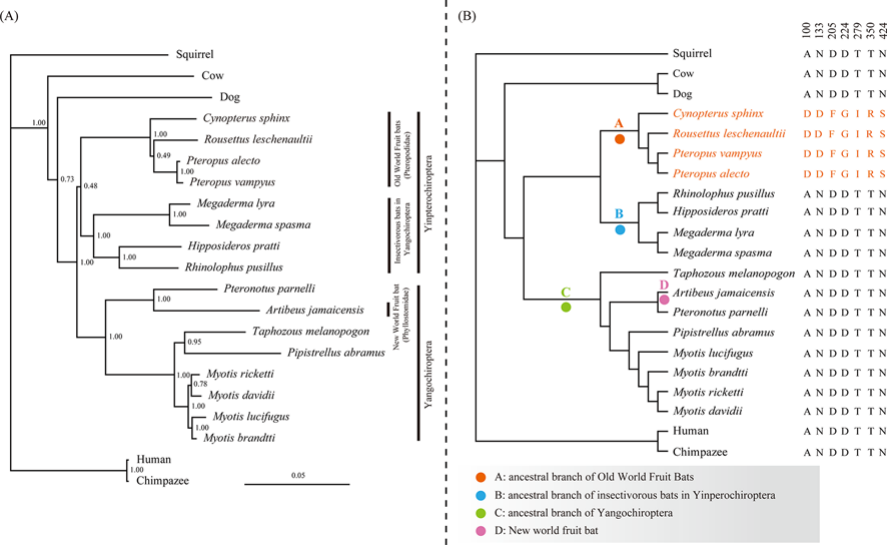
Unconstrained *Nrf2* Bayesian phylogenetic tree and species tree. (A) Unconstrained Bayesian phylogenetic tree based on *Nrf2* coding sequences, under the GTR+G model. Values on the nodes are posterior probabilities. (B) Species tree of the 21 mammals used in this study based on their accepted relationships. Four branches tested by the two-ratio model tests and the branch-site model tests are marked as A, B, C, and D. The four species with orange labels are Old World fruit bats. The seven amino acid substitutions specific to Old World fruit bats are shown (A100D, N133D, D205F, D224G, T279I, T350R and N424S).

To test the selective pressure on *Nrf2* genes in bat lineages, we estimated the rates of nonsynonymous (dN) and synonymous substitutions (dS) using CODEML from the PAML 4 package [37]. As shown in Fig 1B, we labeled four branches for testing: A, ancestral branch of Old World fruit bats; B, ancestral branch of insectivorous bats in suborders Yinpterochiroptera; C, ancestral branch of Yangochiroptera; and D, ancestral branch of New World fruit bats. We first performed the two-ratio model test, which assumes the focal lineage(s) have a different ω value (d_N_/d_S_) compared with other lineages. First, we tested the ancestral lineage leading to Old World fruit bats (branch A) and the branch leading to New World fruit bats (branch D). In addition, to compare the evolutionary pattern of the *Nrf2* gene between frugivorous bats and insectivorous bats, separate two-ratio model tests were conducted with the ancestral branches for insectivorous bats in suborders Yinpterochiroptera (branch B) and Yangochiroptera (branch C). The one-ratio model served as the null hypothesis for all comparisons, which assumes the ω value is equal among all branches.

To further test the selection pressure on the amino acid sites of *Nrf2* genes, we carried the branch-site model A combined with Bayes empirical Bayes (BEB) estimation [38]. The focal branches in these tests were the ancestors of Old World fruit bats (branch A), New World fruit bat (branch D), and insectivorous bats in the suborders Yinpterochiroptera (branch B) and Yangochiroptera (branch C). The remaining branches were set as background. Four site classes of codons are assumed (class 0, class 1, class 2a, and class 2b), class 0 and class 1 codons are assumed to evolve under purifying selection (0 < ω_0_ < 1) and neutral selection (ω_1_ = 1), respectively. Class 2a and class 2b evolve under purifying selection and neutral selection on the background, respectively, while codons in these classes are combined together, as class 2, and assumed to evolve under positive selection (ω_2_ > 1) on the foreground lineage. We applied test1 and test2 of branch-site model A to these four branches (A, B, C, and D), in which branch-site model A as the alternative hypothesis. In test1, the null hypothesis is the M1a model (Nearly neutral), which allowed two site classes (0 < ω_0_ < 1 and ω_1_ = 1). However, in test2, the modified branch-site model A with ω_2_ fixed as 1 was the null hypothesis. To investigate the distribution of selection pressure on sites along the Nrf2 protein sequence, we performed test2 of branch-site model A to calculate the ω value of each amino acid in the ancestral branches leading to Old World fruit bats (branch A), insectivorous bats of suborders Yinpterochiroptera (branch B) and Yangochiroptera (branch C), and the only species of New World fruit bat (*Artibeus lituratus*) (branch D). The statistic 2*Δℓ* (twice the log likelihood difference between the null and alternative hypothesis models) was compared through likelihood ratio tests (LRTs) with the Chi-square test in PAML. Finally, to gain more information on the seven amino acid substitutions in Old World fruit bats, we used different computational methods to predict the biological impact of these mutations on protein function. Protein Variation Effect Analyzer (PROVEAN) [39] and Sorting Intolerant From Tolerant (SIFT) program [40] were used to determine whether a given substitution is deleterious to protein function based on comparison with homologous amino acid sequences. The threshold was set as -1.5 in PROVEAN and 0.05 in the SIFT program.

### Western Blot Validation

Expression levels of Nrf2 protein and the downstream enzyme catalase (CAT) were investigated, and compared, between frugivorous and insectivorous bats. Proteins from brain, heart, and liver were extracted from four representative bat species: *Myotis ricketti* (yangochiropteran insectivorous bats), *Rhinolophus ferrumequinum* (yinpterochiropteran insectivorous bats) and *Cynopterus sphinx*, and *Rousettus leschenaultia* (frugivorous bats from Old World fruit bats). Three individuals were examined for each species. Proteins from rat tissues were used as a positive loading control. Brain, heart, and liver tissues were homogenized using Precellys 24 grinder (Bertin technologies, France) with lysis buffer (10% Glycerol, 2% SDS, 1.25% 2-beta-mercaptoethanol, 25 mM Tris–HCl, pH 6.8) and then heated at 100°C for 10min after a quick spin. The resulting homogenates were centrifuged at 12000 xg at 4°C for 10 min and supernatants were collected. The protein content of each sample was determined utilizing the Quick Start Bradford protein assay kit (Bio-Rad, USA). Proteins in each sample (40 μg/lane) were separated by 10% SDS-PAGE and transferred to 0.2 μm PVDF membranes (Roche, Switzerland). The PVDF membranes were blocked in blocking solution (5% skim milk and 1% BSA) at 4°C for 12 hours and then reacted with primary antibodies including anti-CAT (1:500) and anti-Nrf2 (1:500). Anti-CAT (21260-1-AP) and anti-Nrf2 (sc-722) were purchased from Proteintech Group, Inc. and Santa Cruz Biotechnology, Inc., respectively. Antibodies were selected based on the ability to combine with the conserved epitopes of proteins in bats and rats. After washing with TBST, PVDF membranes were incubated with secondary antibody and then visualized using Immobilon^TM^ Western Chemiluminescence HRP substrate kit (Millipore, USA). Images were captured with the ImageQuant^TM^ LAS 4000 (GE healthcare Life Science, USA) and bands were quantified with ImageQuant^TM^ TL software (version 7.0). Reversible ponceau staining of the membranes were conducted to examine the relative amount of proteins in each lane [41]. The intensity of each band was normalized to the corresponding ponceau stained protein band. Results are shown as mean ± SD and the statistical significance of the difference in CAT and Nrf2 levels among the different species was determined by one-way ANOVA with *post hoc* Holm-Sidak tests. *P* value < 0.05 were considered statistic significant.

## Results

Our final *Nrf2* sequence dataset contained 21 taxa, including four Old World fruit bats (family Pteropodidae), one New World fruit bat (family Phyllostomidae), eleven insectivorous bats and five other mammals. The alignment of the *Nrf2* sequences spanned 1710 nucleotides (570 amino acids), representing 94.2% of the complete coding region. No insertions, deletions or stop codons, and no evidence for recombination breakpoints were detected in our dataset.

Bayesian phylogenetic reconstruction of the *Nrf2* nucleotide sequences revealed a tree where the main groupings were in agreement with the accepted mammalian species tree (Fig 1). The four Old Word fruit bats (*P*. *vampyrus*, *P*. *alecto*, *C*. *sphinx*, and *R*. *leschenaultii*) grouped with species of the family Rhinolophidae (*R*. *pusillus*), Hipposideridae (*H*. *pratti*) and Megadermatidae (*M*. *lyra* and *M*. *spasma*) to comprise suborder Yinpterochiroptera [Bayesian posterior probability (BPP) = 0.48]. The remaining bat species grouped together to comprise the suborder Yangochiroptera (BPP = 1.00).

From the Nrf2 protein sequence alignment, we identified seven amino acid substitutions (100D, 133D, 205F, 224G, 279I, 350R and 424S) shared by all Old World fruit bat species, while all other species, including insectivorous bats and outgroups, shared a different amino acid residue at these locations (Fig 1B). No similar set of amino acid changes was observed in the New World fruit bat. To trace the evolutionary history of these sites, we used Mesquite 2.74 to reconstruct the ancestral amino acid sequences. The results of the ancestral state reconstruction showed that seven specific amino acid replacements (A100D, N133D, D205F, D224G, T279I, T350R and N424S) occurred on the ancestral branch leading to Old World fruit bats (S1 Fig), and these seven amino acid substitutions were specific to this lineage.

To investigate changes in the selection pressure acting on the *Nrf2* gene in frugivorous and insectivorous bats, several two-ratio model tests were carried out on the four branches labeled A, B, C, and D in Fig 1B, which represent the ancestral branches of Old World fruit bats, insectivorous bats in suborders Yinpterochiroptera, ancestral branch of Yangochiroptera and New World fruit bat, respectively. The results of the two-ratio tests are shown in Table 1. However, a comparison of alternative hypotheses with the null hypothesis indicates that the two-ratio models for branches A, B, C, and D do not fit the data better than the one-ratio model.

**Table 1:** Results of the branch model tests of selection pressure on *Nrf2* in bats.

Model^a^	np^b^	*ℓ*	ω_O_^c^	ω_#_^c^	Model Compared	*2Δℓ*	*P* Value
**0.** One ratio: ω_O_ = ω_A_ = ω_B_ = ω_C_ = ω_D_	41	-7676.65	0.24229	= ω_O_			
**1.** Two ratio: ω_O_ = ω_B_ = ω_C_ = ω_D_, ω_A_	42	-7675.60	0.2376	0.3792	1 vs.0	2.1	0.15
**2.** Two ratio: ω_O_ = ω_A_ = ω_C_ = ω_D_, ω_B_	42	-7676.61	0.2427	0.1951	2 vs.0	0.08	0.79
**3.** Two ratio: ω_O_ = ω_A_ = ω_B_ = ω_D_, ω_C_	42	-7675.21	0.2362	0.3911	3 vs.0	2.88	0.09
**4.** Two ratio: ω_O_ = ω_A_ = ω_B_ = ω_C_, ω_D_	42	-7674.86	0.2498	0.1454	4 vs.0	3.57	0.06

^**a**^See Fig 1B for branch labels.

^**b**^np, number of parameters.

^**c**^ω_#_ (ω_A,_ ω_B,_ ω_C,_ ω_D_) and ω_O_, are the d_N_/d_S_ values for branches A, B, C, D and other branches, respectively.

**Fig 2:**
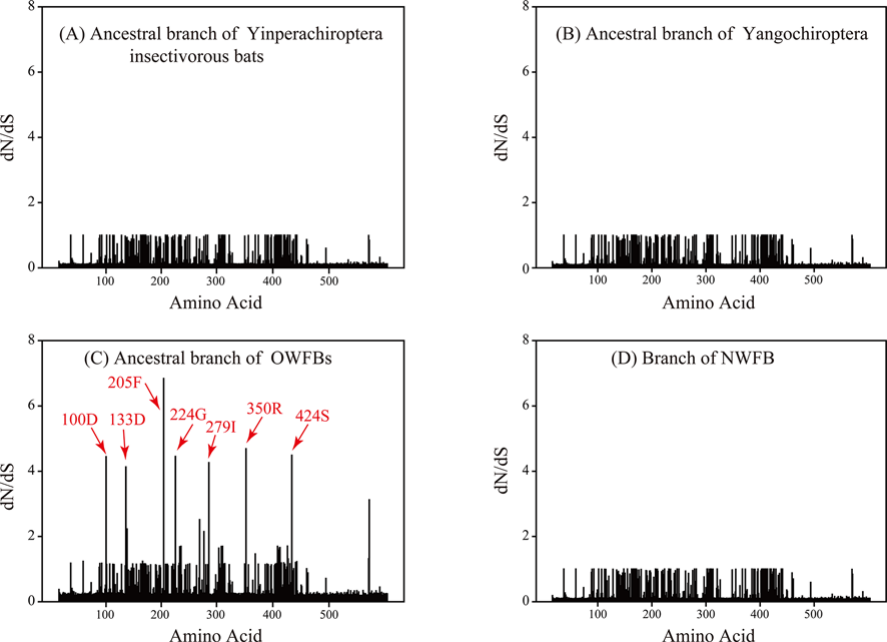
Omega (d_N_/d_S_) values of sites along the Nrf2 protein sequence on selected branches. (A) Ancestral branch of Yinpterochiroptera insectivorous bats, (B) ancestral branch of Yangochiroptera, (C) ancestral branch of Old World fruit bats (OWFBs), and (D) branch of New World fruit bat (NWFB). The red arrows indicate the positions of the seven amino acid replacements in OWFBs with elevated ω values.

To further investigate the distribution of selection pressures on sites along the *Nrf2* sequence, we performed test2 of branch-site model A to calculate the ω values for each amino acid on the ancestral branches leading to the Old World fruit bats (branch A), the insectivorous bats of suborder Yinpterochiroptera (branch B), bats of suborder Yangochiroptera (branch C) and the only species of New World fruit bat in our study (*Artibeus lituratus*) (branch D). Results show that the ω values for amino acid sites on the ancestral branch for Old World fruit bats are higher than that for the other branches (Fig 2). Specifically, the selection pressure for the seven amino acid replacement sites (A100D, N133D, D205F, D224G, T279I, T350R and N424S) were significantly elevated on the ancestral branch leading to Old World fruit bats (branch A) (Fig 2). Moreover, as shown in Table 2, branch-site model A for the ancestral branch of Old World fruit bats did not fit the data better than the branch-site null model which fixed ω_2_ as 1 (2*Δℓ* = 1.88, df = 1, *P* = 0.17; test2), however, branch-site model A for the ancestral branch of Old World fruit bats did fit the data better than M1a model (2*Δℓ* = 7.72, df = 2, *P* = 0.021; test1) (details for test1 and test2 of the branch-site model A can be found in the Materials and Methods Section). Thus, we are unable to reject the possibility of relaxed selection since test1 of branch-site model A cannot distinguish relaxed constraints from positive selection [42]. No evidence for positively selected sites was detected on the branch leading to New World fruit bats (branch D), as the null model cannot be rejected by either test1 or test2 (Table 2). Similar conclusions were also found for the ancestral branches leading to the insectivorous bats of suborder Yinpterochiroptera (branch B) and suborder Yangochiroptera (branch C) (Table 2).

**Table 2:** Results of the branch-site model A tests for the detection of positively selected sites on bats lineages.

Branch-site model^a^	np^b^	Parameters	LRT^c^	*ℓ*	*P*-value	Site with elevated omega values^d^
**M1a (nearly Neutral)**	42	*P*_0_ = 0.771, *P*_1_ = 0.229		-7502.32		
		ω_0_ = 0.063, ω_1_ = 1.00				
**Alternative hypothesis for branch A**	44	*P*_0_ = 0.756, *P*_1_ = 0.224, *P*_2a_ = 0.016, *P*_2b_ = 0.005	Test 1	- 7498.46	**0.021**	100D, 133D, 205F, 224G, 279I, 350R, 424S
		Background: ω_0_ = 0.060, ω_1_ = 1.00, ω_2a_ = 0.060, ω_2b_ = 1.00				
		Foreground: ω_0_ = 0.060, ω_1_ = 1.00, ω_2a_ = 6.962, ω_2b_ = 6.962				
**Null hypothesis for branch A**	43	*P*_0_ = 0.684, *P*_1_ = 0.203, *P*_2a_ = 0.087, *P*_2b_ = 0.026	Test 2	- 7499.40	0.17	Not allowed
		Background: ω_0_ = 0.059, ω_1_ = 1.00, ω_2a_ = 0.059, ω_2b_ = 1.00				
		Foreground: ω_0_ = 0.059, ω_1_ = 1.00, ω_2a_ = 1.00,ω_2b_ = 1.00				
**Alternative hypothesis for branch B**	44	*P*_0_ = 0.771, *P*_1_ = 0.229, *P*_2a_ = 0.000, *P*_2b_ = 0.000	Test 1	- 7502.32	1.0	Not allowed
		Background: ω_0_ = 0.634, ω_1_ = 1.00, ω_2a_ = 0.634, ω_2b_ = 1.00				
		Foreground: ω_0_ = 0.634, ω_1_ = 1.00, ω_2a_ = 1.00, ω_2b_ = 1.00				
**Null hypothesis for branch B**	43	*P*_0_ = 0.771, *P*_1_ = 0.229, *P*_2a_ = 0.000, *P*_2b_ = 0.000	Test 2	- 7502.32	1.0	Not allowed
		Background: ω_0_ = 0.634, ω_1_ = 1.00, ω_2a_ = 0.634, ω_2b_ = 1.00				
		Foreground: ω_0_ = 0.634, ω_1_ = 1.00, ω_2a_ = 1.00, ω_2b_ = 1.00				
**Alternative hypothesis for branch C**	44	*P*_0_ = 0.771, *P*_1_ = 0.229, *P*_2a_ = 0.000, *P*_2b_ = 0.000	Test 1	-7502.32	1.0	Not allowed
		Background: ω_0_ = 0.634, ω_1_ = 1.00, ω_2a_ = 0.634, ω_2b_ = 1.00				
		Foreground: ω_0_ = 0.634, ω_1_ = 1.00, ω_2a_ = 1.00, ω_2b_ = 1.00				
**Null hypothesis for branch C**	43	*P*_0_ = 0.771, *P*_1_ = 0.229, *P*_2a_ = 0.000, *P*_2b_ = 0.000	Test 2	-7502.32	1.0	Not allowed
		Background: ω_0_ = 0.634, ω_1_ = 1.00, ω_2a_ = 0.634, ω_2b_ = 1.00				
		Foreground: ω_0_ = 0.634, ω_1_ = 1.00, ω_2a_ = 1.00, ω_2b_ = 1.00				
**Alternative hypothesis for branch D**	44	*P*_0_ = 0.771, *P*_1_ = 0.229, *P*_2a_ = 0.000, *P*_2b_ = 0.000	Test 1	- 7502.32	1.0	Not allowed
		Background: ω_0_ = 0.634, ω_1_ = 1.00, ω_2a_ = 0.634, ω_2b_ = 1.00				
		Foreground: ω_0_ = 0.634, ω_1_ = 1.00, ω_2a_ = 1.00, ω_2b_ = 1.00				
**Null hypothesis for branch D**	43	*P*_0_ = 0.771, *P*_1_ = 0.229, *P*_2a_ = 0.000, *P*_2b_ = 0.000	Test 2	- 7502.32	1.0	Not allowed
		Background: ω_0_ = 0.634, ω_1_ = 1.00, ω_2a_ = 0.634, ω_2b_ = 1.00				
		Foreground: ω_0_ = 0.634, ω_1_ = 1.00, ω_2a_ = 1.00, ω_2b_ = 1.00				

^a^See Fig 1B for branch labels.

^b^np, number of parameters.

^c^LRT, likelihood ratio test.

^d^location of sites with elevated omega values detected by branch-site model A test refer to *Homo sapiens* sequence.

The sites with BEB posterior probability>0.05 were highlighted by underline.

**Fig 3:**
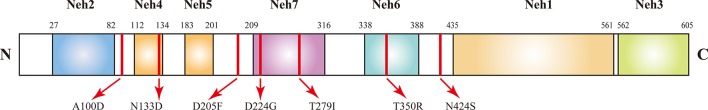
Distribution of the seven OWFBs-specific amino acid replacements in the secondary structure of Nrf2 protein. The locations of the seven amino acid replacements in OWFBs are labeled in red line. The seven Neh domains are shown, Neh1 to Neh7. Neh1 domain (residue 435–561) is the CNC-bZIP domain. Neh2 domain (residue 27–82) is a redox-sensitive regulatory domain that binds with the repressor Keap1, and also contains one of the three nuclear localization sequences (NLS: residue 42–53, 494–511 and 587–593). Neh3 (residue 562–605), Neh4 (residue 112–134) and Neh5 (residue 183–201) domains are involved in Nrf2 transactivation activity. Neh6 domain (residue 338–388) is involved in Nrf2 stability. Neh7 domain (residue 209–316) is involved in interactions with RXRα and functions as a transcriptional repressor.

To test the potential influence of the seven amino acid substitutions on the function of Nrf2 protein in Old World fruit bats, we mapped these sites to the predicted secondary structure of the Nrf2 protein established in previous studies (Fig 3) [43, 44]. Nrf2 belongs to the CNC family of transcription factors and contains seven functional Neh domains, namely, Neh1 to Neh7 [43, 44]. Four of the seven amino acid replacements that occurred in Old World fruit bats locate to Neh domain (Fig 3). The N133D replacement occurs within the Neh4 domain, D224G and T279I are located in the Neh7 domain and T350R is located in Neh6 domain (Fig 3). The remaining three replacements (A100D, D205F, and N424S) are located in sequences between Neh domains in Nrf2 that are not associated with specific functions. To further investigate whether the seven amino acid substitutions potentially affect protein function, we predicted the functional consequences of the substitutions using PROVEAN software and the SIFT program. PROVEAN predicts that three of the seven replacements (D205F, T279I and T350R) were deleterious to Nrf2 protein function (score threshold is -1.3), with the remaining four changes predicted as neutral (Table 3). SIFT suggested that two of the seven replacements (D205F and T350R) were predicted to affect protein function (threshold is 0.05) (Table 3). These results suggest that replacements D205F and T350R are most likely damaging, as they were predicted to be deleterious by both programs.

**Table 3:** PROVEAN and SIFT predictions of the functional consequences of the Nrf2 amino acid replacements in Old World fruit bats.

No.	Variant in Nrf2 protein	Domain	PROVEAN predictions		SIFT predictions	
			PROVEAN Score	Predicted effects on Nrf2 protein (cutoff = -1.3)^a^	SIFT Score	Predicted effects on Nrf2 protein (cutoff = 0.05)^b^
1	A100D	—	-0.600	Neutral	0.32	Neutral
2	N133D	Neh4	-0.936	Neutral	0.33	Neutral
3	D205F	—	-3.378	Deleterious	0.01	Deleterious
4	D224G	Neh7	0.047	Neutral	0.30	Neutral
5	T279I	Neh7	-1.797	Deleterious	0.14	Neutral
6	T350R	Neh6	-1.826	Deleterious	0.01	Deleterious
7	N424S	—	0.314	Neutral	0.44	Neutral

^a^ Positions with PROVEAN Score less than -1.3 are predicted to be deleterious, while those greater than -1.3 are neutral.

^b^ Positions with SIFT Score less than 0.05 are predicted to be deleterious, while those greater than 0.05 are neutral.

**Fig 4:**
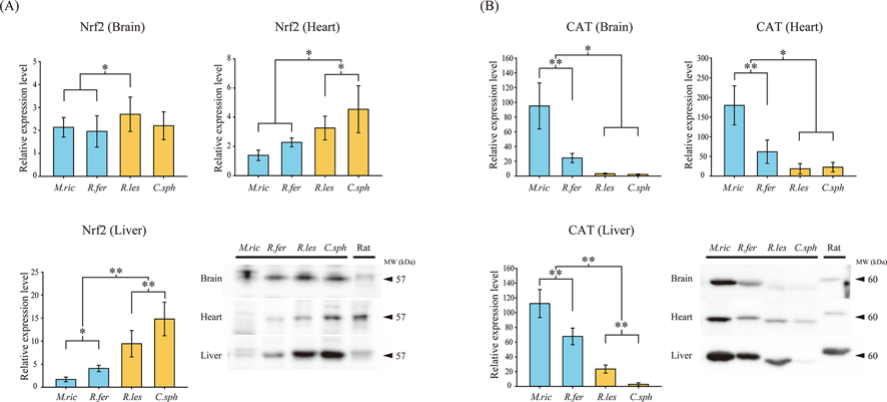
The levels of Nrf2 protein and catalase (CAT) in the brain, heart and liver of four bats species. Four bats species were used in this study and are two insectivorous bats (*M*.*ric*: *Myotis ricketti*; *R*.*fer*: *Rhinolophus ferrumequinum*) and two Old World fruit bats (*R*.*les*: *Rousettus leschenaultia*; *C*.*sph*: *Cynopterus sphinx*). Protein level of (A) Nrf2 protein and (B) catalase in representative bat species and rats (positive control) were determined by Western blotting. Relative levels are presented as mean ± SD. Arrows indicate the predicted molecular weights (kDa) of the proteins. Statistical significance of the differences among species were determined by one-way ANOVA with *post hoc* Holm-Sidak tests: *P < 0.05, **P < 0.001.

To further investigate, and compare, the functional effects of the amino acid replacements between Old World fruit bats and insectivorous bats, we first examined the amount of Nrf2 protein in two representative species of Old World fruit bats (*C*. *sphinx*, *R*. *leschenaultia*) and two insectivorous bats (*M*. *ricketti* from suborder Yangochiroptera; *R*. *ferrumequinum* from Yinpterochiroptera) and the omnivorous rat as the positive loading control. Brain, heart and liver tissue from each species were examined since these three organs are the most vulnerable tissues to oxidative stress [45–47]. In the brain, level of Nrf2 protein in *R*. *leschenaultia* was significant higher than in the two insectivorous bats (*P* < 0.05, one way ANOVA, Holm-Sidak *post hoc* test) (Fig 4A). However, no significant difference was found among *C*. *sphinx*, *M*. *ricketti* and *R*. *ferrumequinum*. In the heart, the amount of Nrf2 protein in the two Old World fruit bats was significantly higher compared to the two insectivorous bats (*P* < 0.05, one way ANOVA, Holm-Sidak *post hoc* test) (Fig 4A). The level of Nrf2 in the heart of *C*. *sphinx* was higher than in *R*. *leschenaultia*, with no significant difference found between *M*. *ricketti* and *R*. *ferrumequinum*. The same situation was observed in the liver, with significantly higher levels of Nrf2 protein in the two Old World fruit bats compared with the two insectivorous bats (*P* < 0.001, one way ANOVA, Holm-Sidak *post hoc* test) (Fig 4A). Moreover, higher levels of Nrf2 protein were found in the livers of *C*. *sphinx* and *R*. *ferrumequinum* in comparison with *R*. *leschenaultia* and *M*. *ricketti*, respectively (*P* < 0.001 and *P* < 0.05 in two comparisons, one way ANOVA, Holm-Sidak *post hoc* test) (Fig 4A).

To determine whether the elevated levels of Nrf2 protein in the three tissues of the Old World fruit bats resulted in enhanced transcriptional function, we conducted Western blot assays to examine the expression of a down-stream enzyme CAT, which is regulated by Nrf2 [48]. However, in contrast to expectations based on the elevated levels of Nrf2, our results showed that the amount of CAT in the brain, heart and liver of the two Old World fruit bats were remarkably lower than in the two insectivorous bats (Fig 4B). In the three tissues from the insectivorous bats, CAT levels were significantly higher in *M*. *ricketti* compared to *R*. *ferrumequinum* (*P* < 0.001, one way ANOVA, Holm-Sidak *post hoc* test) (Fig 4B). The level of Nrf2 in the liver of *R*. *leschenaultia* was significantly higher than in *C*. *sphinx* (*P* < 0.001, one way ANOVA, Holm-Sidak *post hoc* test) (Fig 4B). These results suggest that despite higher levels of Nrf2 protein in Old World fruit bats, the downstream antioxidant enzyme CAT was significantly lower in the three tissues than in insectivorous bats suggesting that the Old World fruit bat Nrf2 sequence have lower transcriptional activity.

## Discussion

In this study, we examined the evolutionary history of the *Nrf2* gene, a central transcriptional regulator of antioxidant enzymes in mammals. Antioxidant enzymes are an important part of the antioxidant defense system, together with low molecular weight antioxidants (LMWAs) [4]. As the daily diet of frugivorous bats contains fruits that are high in LWMAs such as vitamins C and E [23, 24], the abundance of LMWAs might lead to a reduced need for enzymatic antioxidants for free radical scavenging compared with insectivorous bats. *Nrf2*, as the gene encoding a transcriptional regulator of antioxidant enzyme genes, thus might have experienced relaxed constraint in the frugivorous bats. Comparing *Nrf2* sequences among frugivorous and insectivorous bats provides the first data on the molecular adaptation of the *Nrf2* gene in frugivorous bats. Phylogenetic reconstruction analyses showed that the *Nrf2* gene tree is consistent with the accepted species tree (Fig 1) and the d_N_/d_S_ ratio calculated by PAML on each branch was quite low (data not shown), results indicating that the *Nrf2* sequences are evolving conservatively under purifying selection.

Interestingly, alignment of the 21 Nrf2 protein sequences in our dataset identified seven amino acid replacements that were specifically found exclusively in the four Old World fruit bat species (Fig 1B). Ancestral state reconstruction using Mesquite 2.74 showed that all seven amino acid changes occurred precisely on the ancestral branch leading to Old World fruit bats (S1 Fig). Although the results of test1 in branch-site model A for Old World fruit bats revealed that the codons for the seven amino acid replacements had elevated ω value, we cannot distinguish whether these elevated ω values are the result of relaxed selection or positive selection acting on *Nrf2* since test1 was unable to distinguish relaxed constraints from positive selection [42]. In addition, although the ω values for amino acid sites on the ancestral branch for Old World fruit bats are higher than for other branches, the ω value for the ancestral branch leading to Old World fruit bats was 0.387 (data not shown), which is far below the standard for positive selection (ω >> 1). The results of an explicit test for positive selection (test2) was negative, which indicates that there is no strict evidence for positive selection on the *Nrf2* sequences on the ancestral branch for Old World fruit bats (Table 2). Based on the fact that the frugivorous bats get an abundance of LWMAs from their daily diets, their demands for enzymatic antioxidants, which are regulated by *Nrf2*, should be reduced. We hypothesize that the seven amino acid replacements observed in the Old World fruit bat Nrf2 sequences have a functional consequence. To confirm this hypothesis, we performed a biochemical experiment to indirectly investigate the functional activity of Nrf2 by examining the level of the enzyme catalase (CAT), which is a downstream transcriptionally regulated target of Nrf2 [48].

We examined the amount of CAT in three tissues, brain, heart and liver, as these tissues are most vulnerable to oxidative stress [45–47]. Our results showed that CAT level was significantly lower in the brain, heart and liver of the two Old World fruit bats compared to the two insectivorous bats (Fig 4B). It might be assumed that the lower levels of CAT are a consequence of reduced Nrf2 expression in Old World fruit bats, however, this possibility can be ruled out as our biochemical results show that Nrf2 protein levels are significantly higher in the heart and liver of the two Old World fruit bats compared with the two insectivorous bats (Fig 4A). CAT is a major enzymatic antioxidant in aerobic organisms that decomposes hydrogen peroxide to prevent the generation of hydroxyl radical [49], the highly mobile and chemically most reactive species of activated oxygen [50]. Several studies have suggested that *Nrf2* knockout mice are extremely susceptible to oxidative damage and have downregulated expression of CAT [9, 51]. Moreover, *Cat* deficient mice were vulnerable to impairment of brain mitochondria [49]. Thus, the expression level of CAT should represent some of the functions of the transcription factor Nrf2. Combining the results of our molecular evolutionary analysis and biochemical assays, the changes in selection pressure observed at these seven amino acid sites likely are the main cause of the functional change in Nrf2 seen in Old Word fruit bats.

Upregulated Nrf2 expression in these three tissues of Old World fruit bats could also be explained as a compensatory adaptation of the organisms. If the seven amino acid replacements seen in the Old World fruit bat sequences affect Nrf2 protein function, then the increased levels of Nrf2 protein might represent an attempt to compensate for their normal functions [52]. However, although the level of Nrf2 in Old World fruit bats is upregulated, the level of the downstream enzyme CAT is still markedly lower than in insectivorous bats (Fig 4), suggests that upregulation did not completely attenuate the Nrf2 function in Old World fruit bats.

Although the results of the two-ratio test on the ancestral branch leading to Old World fruit bats did not fit the data better than the one-ratio test, we are unable to conclude that *Nrf2* has undergone purifying selection, as the ω value calculated by the branch model is the average of nonsynonymous and synonymous rates over all sites in the protein sequence and over all evolutionary history [53–55]. In order to maintain the function of a protein, most amino acid sites are highly conserved, which results in the signal of elevated ω values at specific sites being overwhelmed by the ubiquitous purifying selection occurring at the vast majority of other sites [54]. Alignment of the protein sequences in our dataset indicates that most amino acid sites in the Nrf2 protein are conserved throughout mammals, thus, the two-ratio test for the ancestral branch of Old World fruit bats was unable to reject the possibility of one-ratio test even when the seven amino acid replacements, with elevated ω values, occurred in Old World fruit bats (Tables 1 and 2).

To further investigate the potential impact of the seven amino acid substitutions on the function of Nrf2 protein in Old World fruit bats, we mapped these substitutions to the secondary structure of the Nrf2 protein (Fig 3) [43, 44]. Nrf2 belongs to the CNC family of transcription factors and contains seven functional Neh domains [43, 44]. Three of the seven amino acid substitutions (D205F, T279I and T350R) in Old World fruit bats are predicted deleterious to Nrf2 function by PROVEAN, with two of these substitutions (D205F and T350R) predicted to be deleterious by the SIFT program (Table 3). The D205F replacement is not located in any of the seven characterized Neh domain, but this substitution is a radical change with the charged and polar amino acid aspartic acid (D) that prefers to be exposed to aqueous environment replaced by phenylalanine (F), which prefers to bury in protein hydrophobic core [56]. This D205F replacement, thus might lead to a change in the structure of Nrf2 that impacts function or cellular localization. The second substitution that was predicted to be deleterious by both methods, T350G, is located in the redox-insensitive Neh6 domain, a domain that is essential for the stability of Nrf2 [57], thus this substitution might affect this function. The T279I replacement, which was predicted to be damaging by PROVEAN but not SIFT, is located in the Neh7 domain of Nrf2, a domain that allows Nrf2 to interact with RXRα and to function as a repressor of ARE-driven gene expression [44], thus could have a functional impact. In support of this, the T279I replacement in Nrf2 is adjacent to residue 278, a residue with a missense mutation in COSMIC database of cancer-associated mutations [58], suggesting that changes near this site have functional consequences. Together, the results of our biochemical assessment of Nrf2 and CAT protein levels, and the predicted deleterious consequences of some replacements in the Nrf2 sequences of Old World fruit bats, suggests that Old World fruit bats produce more Nrf2 protein than insectivorous bats, but that high level of Nrf2 lead to lower amounts of CAT protein (Fig 4). The data suggest that the amino acids replacements, which occurred during the evolution of *Nrf2*, might have yielded an Nrf2 protein that has reduced transcriptional activity that cannot be fully compensated by upregulation. Additional detailed studies are needed in Old World fruit bats to provide direct evidence and understand the consequences of these substitutions on Nrf2 function and structure, which may yield results that are informative for understanding human diseases.

Although New World fruit bats and Old World fruit bats have similar food habits, we did not find evidence of similar evolutionary forces acting on the Nrf2 gene in New World fruit bats (Table 2). None of the seven amino acid replacements observed in the Old World fruit bats were detected in the New World fruit bat (Fig 1B). This evolutionary discrepancy between Old World fruit bats and New World fruit bats can be explained in three ways. First, the Old World fruit bats evolved their frugivorous diets earlier than New World fruit bats (at least 28 mya compared with nearly 20 mya) [34], therefore, New World fruit bats may still retain normal Nrf2 protein function due to their shorter period of time to accumulate sequence changes. Second, it is possible that the retained Nrf2 function in New World fruit bats is due to their divergence from their insectivorous ancestor in the family Phyllostomidae [59]. Third, sequence changes may have occurred in the regulatory region of the Nrf2 gene in New World fruit bats instead of the coding regions. It is noteworthy that several other recent molecular evolution studies on diet-related genes in bats have also found similar evolutionary discrepancies between Old and New World fruit bats [60–63]. For example, PCK1, which plays an important role in glucose homeostasis during fasting, also has specific amino acid changes in the Old World fruit bats but not in New World fruit bats [62]. The *tat* gene, which encodes tyrosine aminotransferase (TAT) that is involved in amino acid metabolism, has undergone relaxed evolution in Old World fruit bats but not in New World fruit bats [63]. Since our results were obtained from a single New World fruit bat of the family Phyllostomidae, more sampling of New World fruit bats is needed to further confirm this finding.

In addition to its critical role in regulating antioxidant enzymes, Nrf2 is also involved in many other cytoprotective pathways that are associated with the aging process [64]. Our results suggest that Nrf2 function is attenuated in Old World fruit bats but maintained in insectivorous bats. Some studies have found that frugivorous bats have shorter lifespans than insectivorous bats [65, 66]. Since there is a close association between oxidative stress and longevity [13–15], it is possible that changes in chiropteran longevity are associated with changes in the selective constraint on *Nrf2*. However, further studies are needed to reveal correlations between *Nrf2* function and chiropteran longevity.

In conclusion, our molecular evolutionary study combined with biochemical characterization shows that *Nrf2* might have experienced relaxed constraint in Old World fruit bats, however, we cannot rule out the possibility of positive selection. The amino acid replacements that occurred during the evolution of *Nrf2* might have functional effects on the Nrf2 protein in Old World fruit bats, potentially due to their LMWAs-rich diets. Our study provides evidence of how diet habit influences metabolic evolution in mammals [67], and might also provide novel insight and more information into understanding chiropteran longevity. Additional in-depth studies are needed to investigate the mechanisms of how these amino acid replacements influence Nrf2 protein function in Old World fruit bats, and we speculate that additional research will discover more genes associated with antioxidant regulation in frugivorous bats that have experienced changes in selection pressure due to their LMWAs-rich diets.

## Supporting Information

S1 Fig Species tree of the 21 mammals with Old World fruit bats specific amino acid substitutions identified by ancestral sequence reconstruction using the maximum parsimony method. (A) A100D, (B) N133D, (C) D205F, (D) D224G, (E) T279I, (F) T350R and (G) N424S. Branch lengths are not shown.
(TIF)

S1 Table *Nrf2* genes with taxa information. (DOCX)

S2 Table Primers used for the amplification of *Nrf2* by PCR. (DOCX)
