# Supplementary figures and images for: Molecular Evolution of the Nuclear Factor (Erythroid-Derived 2)-Like 2 Gene Nrf2 in Old World Fruit Bats (Chiroptera: Pteropodidae)
Source: PLoS One. 2016 Jan 6;11(1):e0146274. doi: 10.1371/journal.pone.0146274 (PMC4703304; doi:10.1371/journal.pone.0146274)

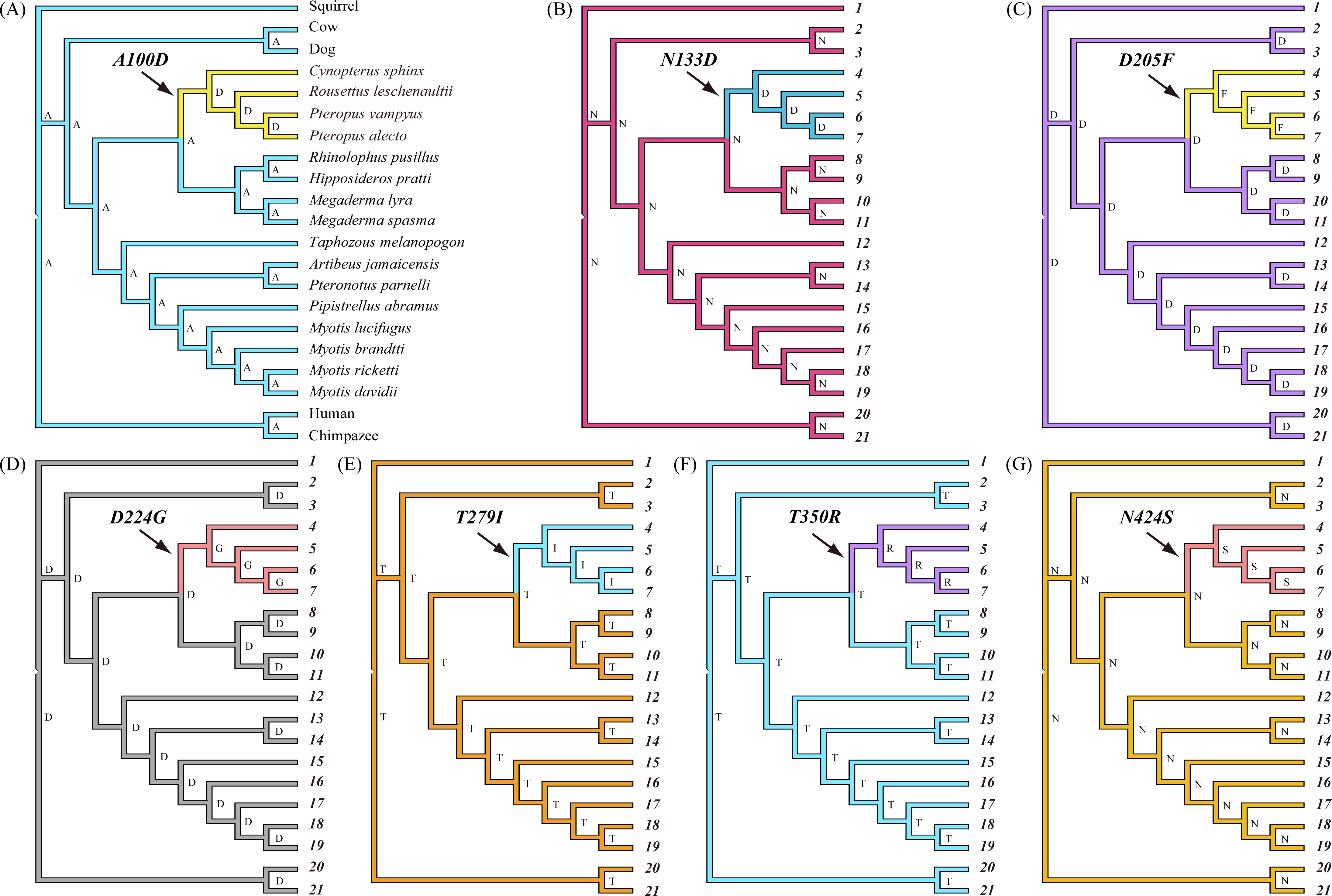

Supplement: S1 Fig — (A) A100D, (B) N133D, (C) D205F, (D) D224G, (E) T279I, (F) T350R and (G) N424S. Branch lengths are not shown. (TIF) [file pone.0146274.s001.tif]
