# Supplementary material for: Molecular Evolution of the Nuclear Factor (Erythroid-Derived 2)-Like 2 Gene Nrf2 in Old World Fruit Bats (Chiroptera: Pteropodidae)
Source: PLoS One. 2016 Jan 6;11(1):e0146274. doi: 10.1371/journal.pone.0146274 (PMC4703304; doi:10.1371/journal.pone.0146274)
Supplement: S1 Table — (DOCX) [file pone.0146274.s002.docx]

| **Species names** | **Family** | **Genus** | **Location** | **Gene Accession Numbers** |
| --- | --- | --- | --- | --- |
| *Cynopterus sphinx* | Pteropodidae | Cynopterus | Zhongshan park (21°280N, 109°70E), Nov.2009, Guangxi province, China | KT345716 |
| *Rousettus leschenaultii* |  | Rousettus | Jinlun cave (23°330N, 108°150E), Nov.2009, Guangxi province, China | KT345715 |
| *Pteropus vampyus* |  | Pteropus | --- | ENSPVAT00000010381 |
| *Pteropus alecto* |  |  | --- | XM_006921233 |
| *Artibeus jamaicensis* | Phyllostomidae | Artibeus | Apr. 2010 in Mexico | KT345722 |
| *Pteronotus parnelli* | Mormoopidea | Pteronotus | Apr. 2010 in Mexico | KT345721 |
| *Myotis ricketti* | Vespertilionidae | Myotis | A cave (28°40N, 116°580E), Nov.2012, Jiangxi province, China | KT345725 |
| *Myotis brandtti* |  |  | --- | XM_005857614 |
| *Myotis davidii* |  |  | --- | XM_006760582 |
| *Myotis lucifugus* |  |  | --- | ENSMLUT00000003908 |
| *Pipistrellus abramus* |  | Pipistrellus | A park (23°260N, 111°300E) in Jul.2011, Jiangxi province, China | KT345724 |
| *Hipposideros pratti* | Hipposideridae | Hipposideros | Tianzidi cave (29°460N, 119°210E), Apr.2013, Zhejiang province, China | KT345719 |
| *Rhinolophus pusillus* | Rhinolophidae | Rhinolophus | Lianhua cave (36°500N, 113°540E), Apr.2013, Hebei province, China | KT345720 |
| *Megaderma lyra* | Megadermatidae | Megaderma | Huong Hoa Nature Reserve (16°56'15N 106°34'52E), May. 2007, Vietnam | KT345717 |
| *Megaderma spasma* |  | Megaderma | Seima Biodiversity Conservation Area (12°15'44N 107°03'49E), Jan.2006, Cambodia | KT345718 |
| *Taphozous melanopogon* | Emballonuridae | Taphozous | Luocongyan (23°14'43N,109°55'11E), Jun.2006, Guangxi province, China | KT345723 |
|  |  |  |  |  |
| *Homo sapiens* | Homonidae | Homo | --- | ENSG00000116044 |
| *Pan troglodytes* |  | Pan | --- | ENSPTRG00000012677 |
| *Canis familiaris* | Canidae | Canis | --- | ENSCAFG00000013506 |
| *Bos taurus* | Bovidae | Bos | --- | ENSBTAG00000019255 |
| *Ictidomys tridecemlineatus* | Sciuridae | Ictidomys | --- | ENSSTOG00000000313 |

**S1 Table. *Nrf2* genes with taxa information**
