# Supplementary material for: Molecular Evolution of the Nuclear Factor (Erythroid-Derived 2)-Like 2 Gene Nrf2 in Old World Fruit Bats (Chiroptera: Pteropodidae)
Source: PLoS One. 2016 Jan 6;11(1):e0146274. doi: 10.1371/journal.pone.0146274 (PMC4703304; doi:10.1371/journal.pone.0146274)
Supplement: S2 Table — (DOCX) [file pone.0146274.s003.docx]

**S2 Table.** **Primers used for the amplification of *Nrf2* by PCR**

|  | **Primer sequence** | **Tm (^o^C)** | **Species** |
| --- | --- | --- | --- |
| F1  R1 | 5'- GACATGGACTTGATTGACATACT -3'  5'- TGTTTGCTGCAGGGAGTATTCAC -3' | 55°C | *Cynopterus sphinx* |
|  |  |  | *Rousettus leschenaultii* |
|  |  |  | *Artibeus jamaicensis* |
|  |  |  | *Pteronotus parnelli* |
|  |  |  | *Myotis ricketti* |
|  |  |  | *Pipostrellus abramus* |
|  |  |  | *Hipposideros pratti* |
|  |  |  | *Rhinolophus pusillus* |
|  |  |  | *Megaderma lyra* |
|  |  |  | *Megaderma spasma* |
|  |  |  | *Taphozous melanopogon* |
